# Bilateral Adrenal Histoplasmosis

**DOI:** 10.4274/balkanmedj.galenos.2019.2019.4.104

**Published:** 2019-10-28

**Authors:** Thachanun Porntharukchareon, Sarin Khahakaew, Thitiwat Sriprasart, Leilani Paitoonpong, Thiti Snabboon

**Affiliations:** 1 Department of Medicine, Chulabhorn Medical Center, Bangkok, Thailand; 2 Department of Medicine, Faculty of Medicine, Chulalongkorn University, Bangkok, Thailand; 3 Department of Medicine, Wetchakarunrasm Hospital, Bangkok, Thailand; 4 Excellent Center in Diabetes, Hormone and Metabolism, King Chulalongkorn Memorial Hospital, Thai Red Cross Society, Bangkok, Thailand

An 85-year-old man presented with a 4 kg weight loss and anorexia for 3 months. He had no history of tuberculosis or other major illness. Physical examination revealed no fever or hyperpigmentation. In addition, chest X-ray was normal. A computed tomography scan of the abdomen revealed bilateral adrenal masses: a 2.0×2.9 cm mass in the right adrenal gland and 3.7×4.3 cm mass in the left adrenal gland (Figure 1). Serologic testing for HIV was negative. The morning serum cortisol level was 5.4 μg/dL, followed by an abnormal response to 250 μg adrenocorticotropic hormone stimulation test (peak serum cortisol, 17 μg/dL). The patient was diagnosed with primary adrenal insufficiency, which was confirmed by an elevated adrenocorticotropic hormone level. Computed tomography-guided fine-needle aspiration cytology of the adrenal lesion revealed features of *Histoplasma* spp. (Figure 2). Thereafter, liposomal amphotericin B (180 mg daily) for 2 weeks was administered, followed by oral itraconazole (400 mg daily) for 1 year. A follow-up computed tomography scan after 6 months of treatment showed a slight decrease in the size of the adrenal masses. However, the adrenal function had not recovered. Written informed consent was obtained from the patient for publication of this case report, including accompanying images.

Histoplasmosis is a disease caused by the dimorphic fungus *Histoplasma capsulatum*, endemic to some regions of the world, including Southeast Asia (1). The main route of infection is via the inhalation of spores from soils contaminated with bat and bird droppings. Although most infected patients are asymptomatic, immunocompromized individuals or older people can develop localized or disseminated forms of the disease.

Notably, the adrenal glands are involved in disseminated histoplasmosis, with bilateral adrenal involvement being common. However, the presence of adrenal insufficiency is reported in only 20%-50% cases (2). The computed tomography findings of adrenal histoplasmosis may vary depending on the stage of the disease. Typical findings include bilateral adrenal masses with the preservation of normal outline, peripheral enhancement, and central hypodensity (3). Calcifications can be observed at later stages. However, similar features are also observed in other systemic infections (tuberculosis or other fungal infections), metastatic cancers, adrenal hemorrhage, lymphoma, and pheochromocytoma. Therefore, a percutaneous biopsy or fine-needle aspiration cytology of the mass is necessary to confirm the diagnosis. Histopathological examination of *H. capsulatum* reveals an intracellular small spherical or oval yeast forms surrounded by a clear ring of space. Additional diagnostic tests include tissue culture, antigen detection, and serology. The recommended treatment for the disseminated form, particularly in hospitalized critically-ill patients, is amphotericin B for 1-2 weeks followed by a 12 month course of itraconazole. Milder cases can be treated by a course of itraconazole for 1 year, albeit with constant monitoring of blood levels. Patients with adrenal insufficiency may require steroid replacement, with the reversal of adrenal dysfunction. Disease recurrence is reported in approximately 10%-15% cases after the cessation of treatment; therefore, long-term follow-up is necessary (3).

## Figures and Tables

**Figure 1:**
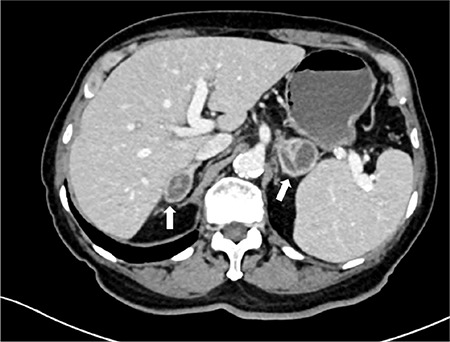
Computed tomography upper abdomen venous phase showing 2.0×2.9 cm mass in the right adrenal gland and 3.7×4.3 cm mass in the left adrenal gland. Both masses (white arrow) show rim enhancement with septation compatible with thin-walled abscess.

**Figure 2:**
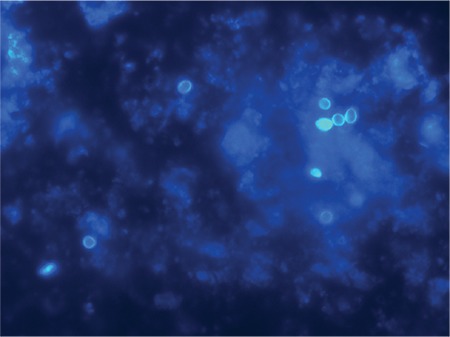
2. Calcofluor-White staining showing round-shaped yeast-like organisms measuring 2-4 μm with narrow-based budding, morphologically compatible with *Histoplasma* spp. (×1000).

## References

[1] Chakrabarti A, Slavin MA (2011). Endemic fungal infections in the Asia-Pacific region. Med Mycol.

[2] Wheat LJ, Azar MM, Bahr NC, Spec A, Relich RF, Hage C (2016). Histoplasmosis. Infect Dis Clin North Am.

[3] Koene RJ, Catanese J, Sarosi GA (2013). Adrenal hypofunction from histoplasmosis: a literature review from 1971 to 2012. Infection.

